# Determinants of the Knowledge of and Attitude towards Tuberculosis in Nigeria

**Published:** 2014-09

**Authors:** K.E. Agho, J. Hall, B. Ewald

**Affiliations:** ^1^School of Science and Health, University of Western Sydney, NSW, Australia; ^2^Centre for Clinical Epidemiology and Biostatistics, University of Newcastle, NSW, Australia

**Keywords:** Attitude, Determinants, Knowledge, Tuberculosis, Nigeria

## Abstract

Globally, Nigeria had the fourth highest incidence of tuberculosis (TB) cases in 2009. Datasets of the 2008 Nigeria Demographic and Health Survey (NDHS) were used for examining factors associated with respondents’ knowledge of and attitude towards TB in Nigeria. With the same age-group of males and females, the sample included 47,193 respondents aged 15-49 years. Factors associated with the knowledge of and attitude towards TB were examined against a set of individual-, household- and community-level variables, using multiple binary logistic regression analyses. Respondents who reported having ever heard of TB was 74.7%. Of those who ever heard of TB, 76.9% believed that TB can be cured, and 19.6% would want a family member's TB to be kept secret. Of those who ever heard of TB, 63.1% believed that TB was spread from person to person through the air by coughing or sneezing. Multivariate analysis indicated that the probability of having poor knowledge of and negative attitude towards TB was consistently significant among the poorest household (lowest wealth quintile), geopolitical regions (North Central), respondents with no schooling, non-working respondents, youngest age-group (15-19 years), and rural areas [adjusted odds ratios (AOR)=0.76, 95% CI 0.66-0.86 for respondents who had ever heard of TB; AOR=0.89, 95% CI 0.80-0.99 for respondents who had ever heard of TB and believed that TB can be cured; AOR=0.83, 95% CI 0.73-0.94 for those who had ever heard of TB and concealed the fact that a family member had TB; and AOR=0.88, 95% CI 0.78-0.99 for those who had ever heard of TB and believed TB was spread from person to person through the air by coughing or sneezing]. Efforts to improve the knowledge of and attitude towards TB in Nigeria should focus on the youngest age-group (15-19 years), the poorest households, and respondents with no schooling. Improving the knowledge and attitude of these groups of individuals may result in an increase in the number of people who will seek early treatment.

## INTRODUCTION

TB remains a major public-health problem in many low-income and middle-income countries (1,2). In 1993, the World Health Organization (WHO) declared TB as a global health emergency (2,3). Since then, there has been an intensification of global efforts to control this disease. Reduction in the burden of TB has been a key priority of the Millennium Development Goal (MDG) 6 agenda (2-4). Although TB is a preventable and curable disease, its incidence increased globally from 9.3 million cases in 2007 to 9.4 million cases in 2008, with most estimated cases occurring in Asia and Africa, owing to the high incidence rate of HIV/AIDS and poor TB case management in these regions (1,5).

Nigeria had the fourth highest rate of TB burden in the world, with an incidence of 0.37-0.55 million in 2008 (1). Nigeria also has the highest number of new TB cases in Africa, with about 300,000 cases recorded each year, resulting in approximately 30,000 deaths annually (1,6,7). To address this problem, substantial investments have been made to the Nigerian Tuberculosis Control Programme (NTCP), which uses the Directly-observed Therapy-Short course (DOTS) to achieve and maintain a high cure-rate (8). Despite these investments, however, early detection of TB cases remains a major obstacle to effective TB case management (6,7). The TB epidemic in Nigeria has also been compounded by the limited number of health staff in the TB control programme (5). For example, the health staff:patient ratio in Nigeria is between 1:160,000 and 1:400,000 (9).

Past studies indicated that patients in Nigeria did not often engage in adequate healthcare-seeking behaviour to reduce the incidence of infectious diseases, including TB, and this has been attributed to the lack of knowledge about the cause, transmission, and significant symptoms of the disease as well as negative social attitudes (10-13). The burden of disease from TB is disproportionally high among individuals living in rural areas, those who live below the poverty line (13,14) as well as in economically-underprivileged countries, highlighting a strong association between poverty and TB (15).

A recent study conducted in Benin-City, Nigeria, indicated that TB awareness programme and knowledge of transmission of TB could increase case-detection rates (7), and individuals who acknowledged the severity of TB were more likely to stay healthy (16). Hence, understanding the factors associated with the knowledge of and attitude towards tuberculosis is considered the first step towards promoting healthcare-seeking behaviour in the community towards infectious diseases in Nigeria (17).

To the best of our knowledge, there has not been any reliable population-based study on the knowledge of and attitude towards TB in Nigeria. Thus, a more detailed understanding of the factors associated with the knowledge of and attitude towards TB is needed to support case detection and reduce TB-related morbidity in Nigeria. The purpose of the present study was to use data from the existing representative survey to identify factors associated with the knowledge of and attitude towards TB in Nigeria.

### Ethical clearance

The DHS project sought and obtained necessary ethical approval from ethics committees in Nigeria before the survey was carried out. Informed consent was obtained from study participants before they were allowed to participate in the survey. The datasets used in this report were completely anonymous with regard to participants’ identity.

## MATERIALS AND METHODS

The present analysis was based on a publicly-available datasets that were collected for the 2008 Nigeria Demographic and Health Survey (NDHS) (18). The survey was conducted by the National Population Commission (19). The NDHS is a useful and valid source of information on the knowledge of and attitude towards TB from a nationally-representative sample of households. The survey sample was selected in two stages. In the first stage, 886 clusters were selected from a list of enumeration areas (EAs) developed from census frame of the 2006 enumeration areas (18). In the second stage, a complete listing of households was carried out in each selected cluster. In total, 34,644 households were selected for the sample, of which 34,070 were successfully interviewed, yielding a response rate of 98%. There was no significant difference between response rates in rural and urban areas (18).

From the sampled households, 33,385 women aged 15-49 years (response rate 96.5%) and 15,486 men aged 15-59 years (response rate 92.6%) were interviewed using a questionnaire to collect data on the respondent's backgrounds, sociodemographic information for all persons usually residing in each household as well as an inventory of household facilities and assets. To answer our research question and ensure that the age-groups were the same for men and women, the current study reports on the 47,193 adults aged between 15 and 49 years.

The explanatory variables were classified into three levels: individual-, household economical- and community-level factors. Individual-level factors included: respondents’ working status, education, age, religion, and marital status. Household-economical-level factors were household wealth index; respondents’ literacy; access to television, magazines/newspapers and radio while community-level factors included type of residence, ethnicity, and geopolitical zones. Geopolitical zones were defined based on ethnic homogeneity of near-perfect political, administrative, and commercial city in Nigeria.

A wealth index was constructed from the data collected through the household questionnaire (20). Using this method recommended by the World Bank Poverty Network and United Nations Children's Fund (20), the data were divided into five categories. The lowest quintile—bottom 20% of the households—was referred to as the poorest households, and the highest quintile—top 20%—as the richest households.

In the remaining part of this paper, TB transmission outcome variables will be expressed as responses to these questions/statements: “Have you ever heard of an illness called TB”; “Can TB be cured?”; “Would you want a family member's TB to be kept secret?”; “TB is spread through air when coughing or sneezing”; and “TB is spread through sharing utensils.” These were expressed as dichotomous variables, with category 1 for ‘yes’ and category 0 for ‘no’. These variables were examined against a set of independent variables (individual-level, household economical- and community-level) to determine factors associated with the knowledge of and attitude towards TB. Analyses were performed using Stata (version 12.0) (StataCorp, College Station, TX, USA). ‘Svy’ commands were employed to allow for adjustments for the cluster-sampling design and the calculation of standard errors. The Taylor series linearization method was used in the surveys when estimating confidence intervals around prevalence estimates of knowledge- and attitude-related questions concerning TB. Multivariate logistic regression was conducted to determine the factors significantly associated with the knowledge of and attitude towards TB. Odds ratios with 95% confidence intervals (CI) were calculated to assess the adjusted risk of independent variables with 95% CI, including 1.0 being regarded as a non-significant result.

## RESULTS

### Characteristics of the sample

As summarized in Table 1, majority of the respondents lived in rural areas (63.7%). Approximately 65% of the respondents were employed in the last 12 months, and 49.4% had attained secondary or higher level of education. Of the total respondents, 70.7% were female. The proportion of individuals who could not read a sentence was 46.3%, and about 24% of the respondents were from the North-West of the country. The majority of respondents were currently married, and 44.7% belonged to the Islamic faith.

### Knowledge of and attitude towards TB: proportion of respondents

Of the total sample of 47,193 adults aged between 15 and 49 years from Nigeria, the proportion of individuals who had ever heard of an illness called TB was 74.7%. The proportion of individuals who reported that TB was curable was 76.9%; 19.6% of individuals reported that TB would be kept secret if a member of their family contracted it among those individuals who thought that TB could be spread from person to person, 63.1% thought that TB could be spread through the air when coughing or sneezing; 36.5% of individuals reported that TB could be spread through sharing utensils; 3.9% of respondents thought TB could be spread through touching a person with TB. The proportions of individuals who thought TB could be spread through food, sexual contact, and mosquito bites were 11%, 5.0%, and 0.8% respectively (Table 2).

### Univariate analysis

Table 3 shows the prevalence estimates of TB transmission (for ever heard of TB, believed that TB can be cured, would want a family member's TB to be kept secret, and TB is spread through coughing or sneezing and sharing utensils) by selected individual-, household economical- and community-level characteristics. It was found that gender, age in categories, respondents’ working status, marital status, frequency of listening to radio, frequency of reading, household wealth, literacy, place of residence, geopolitical zones, ethnicity, and religion were all significantly associated with ever heard of TB, would want a family member's TB to be kept secret, believed that TB can be cured, and TB is spread through coughing or sneezing.

Table 3 indicates that gender, respondents’ working status, marital status, frequency of listening to radio, frequency of reading newspapers or magazines, frequency of watching television, household wealth, educational status, literacy, place of residence, geopolitical zone, ethnicity, and religion were associated with the belief that TB is spread through coughing or sneezing, and TB is spread through sharing utensils.

### Multivariate analysis

Adjusted odds ratios (AORs) were calculated to determine the strength of association between independent variables and ever heard of TB, believed that TB can be cured, and would want a family member's TB to be kept secret as presented in Table 4. Females were significantly less likely to report than male respondents that they had ever heard of TB and believed that TB was curable. Respondents aged between 15 and19 years and those who lived in rural areas were less likely to report that they had ever heard of TB, believed that TB could be cured, and would want a family member's TB to be kept secret compared to those of other age-groups and those who lived in urban areas.

Never-married respondents and those respondents who had limited access to the electronic media were significantly less likely to report that they ever heard of TB and believed that TB is curable. The poorest households (lowest wealth quintiles) were significantly less likely to have heard of TB and believed that TB could be cured than the richest households (highest wealth quintiles). Respondents working in the previous 12 months and those with primary, secondary or higher level of education were significantly more likely to have ever heard of TB, believed that TB was curable, and would want a family member's TB to be kept secret.

**Table 1.** Tuberculosis (TB) by individual, household, economical and community characteristics, Nigeria 2008 (N=47,193)

Characteristics	%	n
Individual-level factors		
Gender		
Male	29.3	13,808
Female	70.7	33,385
Respondent's age (completed years)		
15-19	19.1	9,025
20-24	18.0	8,512
25-29	18.6	8,767
30-34	14.2	6,691
35-39	12.1	5,706
40-44	7.8	3,688
45-49	10.2	4,803
Working status (n=46,676)		
Non-working	35.1	16,400
Working (past 12 months)	64.9	30,276
Educational level		
No education	30.8	14,539
Primary	19.8	9,327
Secondary and above	49.4	23,327
Marital status (n=47,188)		
Never married	31.7	14,945
Currently married	64.8	30,596
Formerly married	3.5	1,647
Religion (n=46,987)		
Catholic	11.6	5,444
Other Christian	42.3	19,866
Islam	44.7	20,999
Traditionalist	1.2	566
Others†	0.2	112
Household-level economic factors		
Wealth index		
Poorest	18.0	8,469
Poorer	18.2	8,566
Middle	18.9	8,910
Richer	21.4	10,101
Richest	23.6	11,147
Frequency of reading newspapers or magazines (n=46,866)		
Almost every day	82.8	38,799
Not at all/less than once a week/at least once a week	17.2	8,067
Frequency of listening to radio (n=47,006)		
Almost every day	62.0	29,135
Not at all/less than once a week/at least once a week	38.0	17,871
Frequency of watching television (n=46,994)		
Almost every day	43.4	20,387
Not at all/less than once a week/at least once a week	56.6	26,607
Literacy (n=46,728)		
Read whole sentences	53.7	25,073
Can't read part/whole sentences	46.3	21,655
Community-level factors		
Place of residence		
Urban	36.3	17,150
Rural	63.7	30,043
Geographical region		
North-Central	14.4	6,812
North-East	12.5	5,907
North-West	23.9	11,259
South-East	11.7	5,539
South-South	16.8	7,910
South-West	20.7	9,766
Ethnicity		
Ekoi	1.6	760
Fulani	5.9	2,764
Hausa	22.4	10,538
Ibibio	2.5	1,159
Igala	1.5	706
Igbo	15.5	7,294
Ijaw/Izon	3.8	1,790
Kanuri/Beriberi	1.9	914
Tiv	2.5	1,163
Yoruba	18.0	8,479
Others‡	24.4	11,464

†Hinduism, Judaism, Grail movement, and Hare Krishnan;

‡Edo, Ebira Nupe, Pyem, Goemai, Kofyar, and other 233 ethno-linguistic groups

The Ekio ethno-linguistic group, when compared with respondents who lived in the other ethno-linguistic groups in Nigeria, were significantly less likely to have heard of TB, believed that TB is curable, and would want a family member's TB to be kept secret. Respondents who belonged to the Catholic faith were more likely to have ever heard of TB, believed that TB could be cured, and would want a family member's TB to be kept secret compared to those who were non-Catholic Christians and others practising religions, such as Hinduism, Judaism, Grail movement, and Hare Krishna faith.

**Table 2.** Responding ‘yes’ to the question about knowledge of TB (N=47,193)

Question	n (weighted)	% (95% CI)
Ever heard of an illness called tuberculosis or TB?		
Yes	35,258	74.7 (73.6-75.8)
Can TB be cured?		
Yes	27,103	76.9 (75.9-77.8)
Would you want a family member's TB to be kept secret?		
Yes	6,895	19.6 (18.7-20.5)
Is TB spread through the air when coughing or sneezing?		
Yes	22,246	63.1 (61.9-64.2)
Is TB spread through sharing utensils?		
Yes	12,852	36.5 (35.2-37.7)
Is TB spread through touching a person with TB?		
Yes	1,361	3.9 (3.5-4.2)
Is TB spread through food?		
Yes	3,874	11.0 (10.3-11.7)
Is TB spread through sexual contact?		
Yes	1,770	5.0 (4.6-5.5)
Is TB spread through mosquito bites?		
Yes	274	0.8 (0.7-0.9)

CI=Confidence interval

Adjusted odds ratios (AORs) of factors that were associated with the belief that TB is spread through coughing or sneezing and sharing utensils are shown in Table 5. Females were significantly less likely to report than males that TB is spread through coughing or sneezing and significantly more likely to report that TB is spread through sharing utensils. Respondents aged between 15 and 19 years were less likely to report that they believed TB is spread through coughing or sneezing and would significantly report that TB was spread through sharing utensils compared to those of other age-groups. Respondents who lived in rural areas were significantly less likely to report that they believed TB is spread through coughing or sneezing compared to those who lived in urban areas.

Non-literate respondents and those respondents who had limited access to the electronic media were significantly less likely to believe that TB is spread through coughing or sneezing and to report that TB is spread through sharing utensils compared to literate respondents and those respondents who had easy access to the electronic media. The poorest households were less likely to believe that TB is spread through coughing or sneezing and to report that TB is spread through sharing utensils compared to the richest households. Respondents working in the previous 12 months and those with primary, secondary or higher levels of education were significantly more likely to believe that TB is spread through coughing or sneezing and to report that TB was spread through sharing utensils than their non-working and uneducated counterparts.

The Ekio ethno-linguistic group, when compared with respondents from other ethno-linguistic groups, were significantly less likely to believe that TB was spread through coughing or sneezing and that TB is spread through sharing cooking-utensils. Respondents who belonged to the Catholic faith were more likely to believe that TB is spread through coughing or sneezing compared to those who were non-Catholic Christians and others practising religions, such as Hinduism, Judaism, Grail movement, and Hare Krishna.

## DISCUSSION

The purpose of this study was to examine the prevalence of and factors associated with the knowledge of and attitude concerning TB in Nigeria. The study indicated that high proportions of respondents were aware of TB and believed that TB is a curable infectious disease. This finding is in agreement with those from a recent study in India (21) and another community-based survey in Ethiopia (22), in which majority of participants in a survey were found to have heard about TB and believed that the disease is curable. Despite this fact, a significantly high number of respondents would conceal the fact that a family member had TB. This meant that most respondents exhibited an unfavourable attitude towards TB patients, which could potentially lead to a stigmatization of these patients. This finding is also consistent with a recent study in Ethiopia (22). Our study also found that a few respondents believed that TB could be spread through sexual contact, food, or a mosquito bite, and by touching a TB patient.

**Table 3.** Prevalence of TB transmission by individual-, household economical- and community-level characteristics

Characteristics	Ever heard of an illness called TB	Believed that TB can be cured	Would want a family member's TB to be kept secret	TB is spread through the air when coughing or sneezing	TB is spread through sharing utensils
% (95% CI)	% (95% CI)	% (95% CI)	% (95% CI)	% (95% CI)
Individual-level factors					
Gender					
Male	83.7 (82.5-84.7)***	86.9 (85.9-87.8)***	17.5 (16.3-18.7)**	71.8 (70.3-73.2)***	31.9 (30.2-33.6)***
Female	71.0 (69.6-72.3)	72.0 (70.8-73.2)	20.6 (19.4-21.8)	58.8 (57.4-60.3)	38.7 (37.3-40.1)
Respondent's age (completed years)					
15-19	61.1 (59.3-62.8)***	73.9 (72.4-75.4)***	24.2 (22.7-25.7)***	61.6 (59.9-63.3)	33.0 (31.3-34.9)***
20-24	73.3 (71.8-74.9)	76.8 (75.4-78.1)	22.1 (20.7-23.5)	62.7 (60.9-64.5)	33.7 (32.1-35.4)
25-29	77.0 (75.6-78.4)	77.4 (76.0-78.8)	19.6 (18.4-20.9)	62.9 (61.3-64.6)	37.5 (35.8-39.2)
30-34	79.1 (77.7-80.5)	78.5 (77.0-79.9)	18.2 (16.9-19.5)	64.3 (62.4-66.0)	37.1 (35.2-39.1)
35-39	80.3 (78.8-81.8)	78.5 (76.9-80.1)	17.1 (15.8-18.5)	63.7 (61.9-65.6)	39.0 (37.1-40.9)
40-44	78.6 (76.8-80.3)	75.6 (73.7-77.5)	18.0 (16.3-19.9)	62.2 (59.9-64.4)	38.3 (36.0-40.5)
45-49	82.7 (81.2-84.1)	77.0 (75.3-78.7)	14.8 (13.5-16.3)	64.4 (62.4-66.4)	38.5 (36.5-40.6)
Working status					
Non-working	66.4 (64.7-67.9)***	74.5 (73.1-75.9)***	21.4 (20.2-22.8)***	60.2 (58.5-61.9)***	33.5 (31.9-35.2)***
Working (past 12 months)	79.3 (78.2-80.4)	78.0 (76.9-79.0)	18.6 (17.7-19.6)	64.5 (63.3-65.7)	37.8 (36.5-39.1)
Educational level					
No education	59.7 (57.8-61.5)***	62.6 (60.7-64.5)***	20.9 (19.2-22.7)	48.4 (46.4-50.5)***	23.3 (21.6-25.2)***
Primary	74.0 (72.5-75.6)	75.2 (73.7-76.6)	18.9 (17.6-20.2)	59.4 (57.7-61.1)	34.6 (32.9-36.2)
Secondary and above	84.3 (83.4-85.3)	83.8 (82.9-84.6)	19.2 (18.2-20.3)	70.9 (69.5-72.1)	42.9 (41.4-44.4)
Marital status					
Never married	75.1 (73.8-76.4)**	82.3 (81.2-83.3)***	21.7 (20.6-22.9)***	68.8 (67.3-70.2)***	38.3 (36.8-39.9)***
Currently married	74.2 (72.8-75.5)	74.2 (73.0-75.3)	18.6 (17.6-19.6)	60.3 (59.0-61.6)	35.3 (34.0-36.7)
Formerly married	80.8 (78.1-83.2)	77.6 (75.0-80.0)	18.2 (15.9-20.7)	62.2 (59.2-65.1)	40.0 (36.8-43.3)
Religion					
Catholic	86.4 (84.5-88.1)***	82.5 (80.7-84.2)***	23.5 (21.5-25.6)***	64.4 (61.6-67.0)***	41.8 (39.0-44.7)
Other Christians	79.3 (77.8-80.7)	81.2 (80.1-82.2)	17.1 (16.0-18.2)	68.3 (66.9-69.7)	44.0 (42.6-45.5)
Islam	67.8 (66.1-69.5)	70.4 (68.7-72.0)	21.1 (19.7-22.5)	56.9 (55.0-58.7)	26.3 (24.6-28.1)
Traditionalist	61.7 (54.9-68.2)	71.7 (66.7-76.2)	19.7 (14.4-26.4)	65.5 (60.0-70.7)	34.9 (28.6-41.8)
Others	73.5 (61.2-82.9)	76.8 (61.6-87.2)	8.0 (4.0-15.5)	65.4 (48.3-79.3)	34.2 (22.8-47.7)
Household-level economic factors					
Wealth index					
Poorest	60.8 (58.5-63.2)***	64.9 (62.4-67.3)***	22.5 (20.5-24.7)***	49.8 (47.5-52.0)***	24.7 (22.5-27.1)***
Poorer	65.8 (63.9-67.7)	70.1 (68.1-72.0)	20.8 (19.1-22.5)	55.1 (52.9-57.4)	28.1 (25.9-30.5)
Middle	73.3 (71.6-75.1)	76.4 (74.9-77.9)	17.2 (15.8-18.8)	60.9 (58.9-62.9)	36.4 (34.3-38.6)
Richer	80.7 (79.2-82.1)	80.9 (79.6-82.1)	17.7 (16.4-19.1)	67.2 (65.4-68.9)	39.6 (37.6-41.6)
Richest	87.8 (86.6-88.8)	84.0 (82.8-85.2)	20.4 (18.9-21.9)	72.8 (71.0-74.5)	44.8 (42.8-46.9)
Frequency of reading newspapers or magazines					
Almost every day	90.9 (90.0-91.8)***	73.7 (72.6-74.7)***	19.9 (18.9-20.8)	59.4 (58.2-60.6)***	34.0 (32.8-35.3) ***
Not at all/less than once a week/at least once a week	71.4 (70.1-72.6)	89.0 (88.0-89.9)	18.5 (17.2-19.9)	77.1 (75.5-78.7)	45.7 (43.7-47.8)
Frequency of listening to radio					
Almost every day	82.1 (81.2-83.0)***	80.9 (80.0-81.7)***	18.6 (17.6-19.5)***	66.7 (65.5-67.9)***	38.1 (36.7-39.5)***
Not at all/less than once a week/at least once a week	62.7 (61.0-64.3)	68.2 (66.6-69.9)	21.6 (20.3-23.1)	55.4 (53.8-56.9)	33.0 (31.5-34.5)
Frequency of watching television					
Almost every day	84.4 (83.4-85.3)***	83.3 (82.4-84.1)***	19.6 (18.5-20.8)	70.7 (69.4-72.0)***	42.6 (41.1-44.2)***
Not at all/less than once a week/at least once a week	67.3 (65.9-68.7)	70.7 (69.4-72.0)	19.5 (18.4-20.6)	55.8 (54.4-57.2)	30.6 (29.1-32.0)
Literacy					
Can read whole sentences	84.3 (83.3-85.2)***	83.4 (82.6-84.2)***	19.2 (18.2-20.2)	70.5 (69.3-71.8)***	42.4 (41.0-43.9)***
Can't read part/whole sentences	63.5 (62.0-65.1)	66.9 (65.3-68.3)	20.2 (18.9-21.6)	52.0 (50.4-53.6)	27.3 (25.9-28.9)
Community-level factors					
Place of residence					
Urban	84.5 (83.1-85.8)***	81.5 (80.3-82.6)***	20.7 (19.3-22.2)*	69.7 (68.1-71.3)***	40.7 (38.6-42.9)***
Rural	69.1 (67.6-70.6)	73.7 (72.4-74.9)	18.7 (17.7-19.9)	58.5 (56.9-60.0)	33.5 (31.9-35.1)
Geographical region					
North-Central	65.4 (61.4-69.1)***	80.8 (78.7-82.7)***	18.2 (15.9-20.8)**	72.1 (69.6-74.4)***	25.1 (22.5-27.9)***
North-East	70.7 (67.1-74.1)	66.9 (63.6-70.0)	22.0 (19.6-24.5)	58.5 (55.3-61.6)	36.6 (33.4-40.0)
North-West	68.7 (66.2-71.1)	71.1 (68.5-73.6)	22.3 (20.2-24.6)	50.9 (47.7-54.1)	18.3 (15.7-21.1)
South-East	91.7 (89.9-93.3)	83.4 (81.5-85.1)	20.3 (18.4-22.3)	59.5 (56.2-62.8)	44.9 (41.8-48.0)
South-South	76.2 (73.5-78.8)	81.0 (78.8-83.0)	19.0 (17.1-21.1)	68.0 (65.5-70.5)	43.8 (41.4-46.2)
South-West	79.7 (77.4-81.8)	78.3 (76.5-80.0)	16.2 (14.3-18.4)	71.0 (68.8-73.2)	49.7 (47.5-51.8)
Ethnicity					
Ekoi	89.7 (86.1-92.4)***	95.2 (93.3-96.6)***	39.7 (35.1-44.3)***	83.1 (79.6-86.1)***	60.9 (56.7-64.9)***
Fulani	55.0 (51.2-58.8)	68.0 (64.6-71.3)	22.6 (19.4-26.2)	45.4 (41.2-49.7)	26.5 (22.8-30.7)
Hausa	70.7 (68.5-72.8)	69.4 (66.9-71.8)	22.8 (20.9-24.9)	51.1 (48.3-53.9)	17.7 (15.8-19.9)
Ibibio	78.5 (74.5-82.1)	85.4 (81.4-88.6)	15.4 (12.3-19.2)	67.1 (61.8-72.0)	38.9 (32.5-45.7)
Igala	55.3 (50.1-60.4)	80.3 (75.0-84.6)	6.1 (4.0-9.4)	77.8 (72.4-82.3)	14.3 (9.5-20.9)
Igbo	89.6 (87.8-91.2)	82.9 (81.3-84.4)	20.3 (18.6-22.0)	62.3 (59.6-64.9)	45.6 (43.0-48.2)
Ijaw/Izon	76.3 (69.2-82.2)	79.7 (75.8-83.2)	13.4 (10.8-16.5)	60.7 (56.3-64.9)	49.1 (44.6-53.7)
Kanuri/Beriberi	65.5 (58.6-71.8)	50.2 (40.8-59.5)	31.5 (25.4-38.4)	59.1 (53.9-64.0)	32.0 (28.7-35.4)
Tiv	89.2 (83.5-93.2)	72.4 (68.1-76.3)	28.8 (23.6-34.6)	64.5 (60.0-68.8)	25.9 (22.4-29.8)
Yoruba	80.5 (78.7-82.3)	78.5 (76.7-80.2)	15.3 (13.5-17.3)	72.1 (69.9-74.2)	45.4 (42.9-47.9)
Others	68.3 (65.8-70.6)	78.6 (76.9-80.3)	17.2 (15.9-18.7)	68.4 (66.6-70.2)	39.1 (37.0-41.2)

*p<0.05;

**p<0.01;

***p<0.001; χ^2^-test was applied to determine statistical significance

**Table 4.** Logistic analysis on those who heard of TB, believed that TB is curable and would want a family member's TB to be kept secret by individual-, household-, economical- and community-level characteristics

Characteristics	Ever heard of an illness called TB				Believing TB can be cured				Would want a family member's TB to be kept secret			
	Unadjusted odds ratio	P	Adjusted odds ratio (AOR)	P	Unadjusted odds ratio	P	Adjusted odds ratio (AOR)	P	Unadjusted odds ratio	P	Adjusted odds ratio (AOR)	P
	OR (95% CI)		AOR (95% CI)		OR (95% CI)		AOR (95% CI)		OR (95% CI)		AOR(95%CI)	
Individual-level factors												
Gender												
Male	1.00		1.00		1.00		1.00		1.00		1.00	
Female Respondent's age (completed years) 15-19	0.48 (0.44-0.52) 1.00	<0.001	0.69 (0.62-0.76) 1.00	<0.001	0.39 (0.35-0.43) 1.00	<0.001	0.46(0.41-0.51) 1.00	<0.001	1.22(1.09-1.36) 1.00	<0.001	1.18 (1.06-1.32) 1.00	0.003
20-24	1.76 (1.63-1.89)	<0.001	1.72 (1.58-1.88)	<0.001	1.18 (1.07-1.30)	0.001	1.24 (1.11-1.38)	<0.001	0.90 (0.82-0.98)	0.021	0.92 (0.84-1.01)	0.089
25-29	2.14 (1.98-2.31)	<0.001	2.23 (2.03-2.45)	<0.001	1.21 (1.10-1.34)	<0.001	1.42 (1.27-1.60)	<0.001	0.78 (0.71-0.85)	<0.001	0.82 (0.74-0.92)	<0.001
30-34	2.39 (2.19-2.61)	<0.001	2.51 (2.25-2.81)	<0.001	1.29 (1.16-1.43)	<0.001	1.58 (1.40-1.78)	<0.001	0.70 (0.63-0.78)	<0.001	0.76 (0.67-0.87)	<0.001
35-39	2.61 (2.38-2.86)	<0.001	2.87 (2.57-3.22)	<0.001	1.31 (1.17-1.46)	<0.001	1.66 (1.45-1.89)	<0.001	0.65 (0.58-0.72)	<0.001	0.75 (0.64-0.87)	<0.001
40-44	2.36 (2.14-2.61)	<0.001	3.18 (2.81-3.60)	<0.001	1.11 (0.99-1.26)	0.081	1.69 (1.46-1.96)	<0.001	0.70 (0.61-0.80)	<0.001	0.62 (0.54-0.72)	<0.001
45-49	3.11 (2.81-3.43)	<0.001	3.85 (3.39-4.37)	<0.001	1.19 (1.07-1.33)	0.002	1.55 (1.35-1.79)	<0.001	0.55 (0.48-0.62)	<0.001	0.62 (0.54-0.72)	<0.001
Working status												
Non-working	1.00		1.00		1.00				1.00			
Working (past 12 months)	1.94 (1.81-2.07)	<0.001	1.46 (1.36-1.57)	<0.001	1.21 (1.12-1.31)	<0.001			0.83 (0.77-0.90)	<0.001		
Educational level												
No education	1.00		1.00		1.00		1.00		1.00			
Primary	1.90 (1.73-2.10)	<0.001	1.57 (1.42-1.73)	<0.001	1.83 (1.65-2.03)	<0.001	1.33 (1.20-1.47)	<0.001	0.89 (0.79-1.01)	0.063		
Secondary and above	3.63 (3.28-4.01)	<0.001	2.37 (1.92-2.91)	<0.001	3.15 (2.86-3.48)	<0.001	1.83 (1.63-2.04)	<0.001	0.91 (0.81-1.02)	0.117		
Marital status													
Never married	1.00		1.00		1.00		1.00		1.00		1.00	
Currently married	0.95 (0.89-1.03)	0.220	1.19 (1.09-1.31)	<0.001	0.62 (0.57-0.67)	<0.001	0.90 (0.81-1.00)	0.040	0.82 (0.76-0.88)	<0.001	0.87 (0.79-0.96)	0.004
Formerly married	1.39 (1.19-1.63)	<0.001	1.32 (1.10-1.57)	0.003	0.76 (0.65-0.88)	<0.001	1.10 (0.92-1.31)	0.303	0.80 (0.68-0.94)	0.008	0.89 (0.75-1.07)	0.211
Religion												
Catholic	1.00		1.00		1.00		1.00		1.00		1.00	
Other Christians	0.60 (0.52-0.71)	<0.001	0.94 (0.80-1.10)	0.417	0.92 (0.81-1.04)	0.161	0.99 (0.87-1.12)	0.874	0.68 (0.60-0.76)	<0.001	0.87 (0.77-0.98)	0.021
Islam	0.33 (0.28-0.39)	<0.001	0.72 (0.60-0.88)	0.001	0.50 (0.43-0.58)	<0.001	0.81 (0.68-0.95)	0.012	0.87 (0.76-1.01)	0.061	1.01 (0.84-1.21)	0.947
Traditionalist	0.26 (0.19-0.35)	<0.001	0.69 (0.53-0.89)	0.005	0.52 (0.41-0.67)	<0.001	0.92 (0.70-1.21)	0.548	0.81 (0.54-1.20)	0.291	1.09 (0.73-1.64)	0.667
Others	0.44 (0.25-0.77)	0.005	0.70 (0.41-1.18)	0.183	0.70 (0.33-1.46)	0.337	0.68 (0.34-1.39)	0.292	0.29 (0.14-0.61)	0.001	0.35 (0.17-0.75)	0.007
Household-level economic factors												
Wealth index												
Poorest	1.00		1.00		1.00		1.00		1.00		1.00	
Poorer	1.25 (1.13-1.38)	<0.001	1.10 (1.00-1.21)	0.044	1.29 (1.14-1.47)	<0.001	1.11 (0.99-1.25)	0.072	0.90 (0.79-1.03)	0.130	0.95 (0.83-1.09)	0.444
Middle	1.78 (1.57-2.01)	<0.001	1.24 (1.10-1.40)	0.001	1.76 (1.54-2.01)	<0.001	1.23 (1.08-1.40)	0.002	0.73 (0.63-0.86)	<0.001	0.77 (0.66-0.91)	0.002
Richer	2.68 (2.35-3.07)	<0.001	1.42 (1.22-1.64)	<0.001	2.34 (2.05-2.68)	<0.001	1.36 (1.17-1.57)	<0.001	0.76 (0.65-0.88)	<0.001	0.79 (0.66-0.94)	0.009
Richest	4.65 (4.03-5.37)	<0.001	1.86 (1.55-2.22)	<0.001	2.91 (2.53-3.35)	<0.001	1.48 (1.24-1.76)	<0.001	0.89 (0.76-1.04)	0.146	0.97 (0.79-1.18)	0.736
Frequency of reading newspapers or magazines											
Almost every day	1.00		1.00		1.00		1.00		1.00			
Not at all/less than once a week/at least once a week	3.99 (3.57-4.48)	<0.001	1.57 (1.40-1.78)	<0.001	2.91 (2.63-3.22)	<0.001	1.44 (1.30-1.61)	<0.001	0.93 (0.84-1.02)	0.108		
Frequency of listening to radio												
Almost every day	1.00		1.00		1.00		1.00		1.00		1.00	
Not at all/less than once a week/at least once a week	0.36 (0.34-0.39)	<0.001	0.59 (0.55-0.63)	<0.001	0.50 (0.46-0.54)	<0.001	0.78 (0.72-0.85)	<0.001	1.21 (1.11-1.32)	<0.001	1.15 (1.05-1.26)	0.003
Frequency of watching television												
Almost every day	1.00				1.00		1.00		1.00		1.00	
Not at all/less than once a week/at least once a week	0.38 (0.35-0.42)	<0.001			0.48 (0.44-0.52)	<0.001	0.90 (0.82-1.00)	0.042	0.98 (0.90-1.08)	0.741	0.87 (0.78-0.96)	0.008
Literacy												
Read whole sentences	1.00		1.00		1.00				1.00			
Can't read part/whole sentences	0.32 (0.30-0.36)	<0.001	0.74 (0.62-0.88)	0.001	0.39 (0.36-0.43)	<0.001			1.05 (0.96-1.16)	0.282		
Community-level factors Place of residence												
Urban	1.00		1.00		1.00		1.00		1.00		1.00	
Rural	0.41 (0.37-0.47)	<0.001	0.76 (0.66-0.86)	<0.001	0.63 (0.57-0.70)	<0.001	0.89 (0.80-0.99)	0.032	0.87 (0.78-0.98)	0.017	0.83 (0.73-0.94)	0.004
Geographical region												
North-Central	1.00		1.00		1.00		1.00		1.00		1.00	
North-East	1.28 (1.01-1.62)	0.044	2.84 (2.27-3.56)	<0.001	0.48 (0.40-0.58)	0.000	0.75 (0.62-0.92)	0.005	1.25 (1.01-1.55)	0.043	1.52 (1.22-1.88)	<0.001
North-West	1.17 (0.85-1.43)	0.135	1.88 (1,52-2.33)	<0.001	0.58 (0.49-0.70)	0.000	0.88 (0.70-1.09)	0.232	1.31 (1.07-1.60)	0.010	1.43 (1.16-1.77)	0.001
South-East	5.89 (4.46-7.78)	<0.001	3.99 (2.79-5.70)	<0.001	1.20 (1.00-1.44)	0.056	0.95 (0.70-1.29)	0.753	1.15 (0.94-1.40)	0.184	1.43 (1.10-1.86)	0.007
South-South	1.69 (1.36-2.11)	<0.001	0.99 (0.79-1.24)	0.918	1.04 (0.86-1.25)	0.710	0.50 (0.39-0.64)	<0.001	1.07 (0.87-1.31)	0.529	1.13 (0.91-1.41)	0.279
South-West	2.09 (1.68-2.59)	<0.001	1.00 (0.82-1.23)	0.965	0.88 (0.74-1.04)	0.124	0.59 (0.46-0.75)	<0.001	0.88 (0.70-1.10)	0.254	1.11 (0.88-1.41)	0.365
Ethnicity												
Ekoi	1.00		1.00		1.00		1.00		1.00		1.00	
Fulani	0.14 (0.10-0.20)	<0.001	0.21 (0.13-0.32)	<0.001	0.11 (0.07-0.15)	<0.001	0.13 (0.08-0.21)	<0.001	0.44 (0.33-0.58)	<0.001	0.43 (0.31-0.60)	<0.001
Hausa	0.28 (0.20-0.39)	<0.001	0.35 (0.23-0.54)	<0.001	0.11 (0.08-0.16)	<0.001	0.11 (0.07-0.17)	<0.001	0.46 (0.36-0.57)	<0.001	0.45 (0.33-0.60)	<0.001
Ibibio	0.40 (0.27-0.60)	<0.001	0.28 (0.18-0.45)	<0.001	0.31 (0.19-0.49)	<0.001	0.23 (0.14-0.38)	<0.001	0.28 (0.20-0.39)	<0.001	0.29 (0.20-0.42)	<0.001
Igala	0.14 (0.10-0.21)	<0.001	0.14 (0.09-0.22)	<0.001	0.20 (0.13-0.31)	<0.001	0.10 (0.06-0.16)	<0.001	0.10 (0.06-0.17)	<0.001	0.10 (0.06-0.17)	<0.001
Igbo	0.99 (0.68-1.45)	0.970	0.27 (0.17-0.42)	<0.001	0.24 (0.17-0.34)	<0.001	0.11 (0.07-0.17)	<0.001	0.39 (0.31-0.49)	<0.001	0.36 (0.29-0.46)	<0.001
Ijaw/Izon	0.37 (0.23-0.61)	<0.001	0.35 (0.22-0.56)	<0.001	0.20 (0.13-0.30)	<0.001	0.16 (0.10-0.24)	<0.001	0.24 (0.18-0.33)	<0.001	0.25 (0.18-0.35)	<0.001
Kanuri/Beriberi	0.22 (0.14-0.34)	<0.001	0.24 (0.15-0.40)	<0.001	0.05 (0.03-0.08)	<0.001	0.07 (0.04-0.11)	<0.001	0.70 (0.49-0.99)	0.045	0.63 (0.43-0.93)	0.019
Tiv	0.94 (0.53-1.68)	0.839	1.30 (0.67-2.50)	0.433	0.13 (0.09-0.20)	<0.001	0.08 (0.05-0.13)	<0.001	0.61 (0.44-0.84)	0.003	0.57 (0.41-0.80)	0.001
Yoruba	0.48 (0.34-0.68)	<0.001	0.30 (0.20-0.46)	<0.001	0.18 (0.13-0.26)	<0.001	0.11 (0.07-0.17)	<0.001	0.28 (0.21-0.36)	<0.001	0.26 (0.20-0.34)	<0.001
Others	0.25 (0.18-0.35)	<0.001	0.21 (0.15-0.31)	<0.001	0.18 (0.13-0.26)	<0.001	0.13 (0.09-0.19)	<0.001	0.32 (0.26-0.40)	<0.001	0.32 (0.25-0.41)	<0.001

AOR=Adjusted odds ratio; CI=Confidence interval; OR=Odds ratio; p=Level of significance

**Table 5.** Logistic analysis of those who thought that TB is caused by coughing or sneezing and through sharing utensils by individual-, household sconomic- and community-level characteristics

Characteristics	TB is spread through							
	air when coughing or sneezing				sharing utensils			
	Unadjusted odds ratio		Adjusted odds ratio (AOR)		Unadjusted odds ratio		Adjusted odds ratio (AOR)	
	OR (95% CI)	p	AOR (95% CI)	p	OR (95% CI)	p	AOR (95% CI)	p
Individual-level factors								
Gender								
Male	1.00		1.00		1.00		1.00	
Female	0.55 (0.51-0.60)	<0.001	0.64 (0.58-0.70)	<0.001	1.34 (1.23-1.46)	<0.001	1.62 (1.47-1.78)	<0.001
Respondent's age (completed years)								
15-19	1.00		1.00		1.00		1.00	
20-24	1.06 (0.97-1.16)	0.181	1.09 (0.99-1.20)	0.061	1.04 (0.95-1.13)	0.433	1.10 (0.99-1.21)	0.053
25-29	1.08 (0.99-1.17)	0.077	1.17 (1.07-1.29)	0.001	1.21 (1.10-1.32)	<0.001	1.32 (1.19-1.47)	<0.001
30-34	1.13 (1.04-1.24)	0.007	1.30 (1.17-1.45)	<0.001	1.19 (1.08-1.31)	<0.001	1.37 (1.22-1.53)	<0.001
35-39	1.13 (1.03-1.24)	0.01	1.34 (1.20-1.50)	<0.001	1.29 (1.17-1.43)	<0.001	1.52 (1.22-1.53)	<0.001
40-44	1.05 (0.9-1.17)	0.367	1.45 (1.28-1.65)	<0.001	1.27 (1.14-1.41)	<0.001	1.56 (1.36-1.77)	<0.001
45-49	1.15 (1.04-1.27)	0.007	1.41 (1.24-1.61)	<0.001	1.28 (1.15-1.42)	<0.001	1.69 (1.48-1.93)	<0.001
Working status								
Non-working	1.00		1.00		1.00		1.00	
Working (past 12 months)	1.20 (1.13-1.29)	<0.001	1.18 (1.11-1.26)	<0.001	1.20 (1.12-1.28)	<0.001	1.17 (1.09-1.27)	<0.001
Maternal education								
No education	1.00		1.00		1.00		1.00	
Primary	1.54 (1.40-1.71)	<0.001	1.11 (0.99-1.23)	0.055	1.70 (1.53-1.89)	<0.001	1.14 (1.02-1.27)	0.023
Secondary and above	2.56 (2.31-2.83)	<0.001	1.22 (1.02-1.46)	0.033	2.45 (2.19-2.74)	<0.001	1.31 (1.09-1.57)	0.003
Marital status							
Never married	1.00		1.00		1.00		1.00	
Cunently married	0.70 (0.65-0.75)	<0.001	0.91 (0.84-0.99)	0.034	0.88 (0.82-0.94)	0.000	0.92 (0.84-0.99)	0.042
Formerly married	0.74 (0.65-0.85)	<0.001	0.96 (0.82-1.12)	0.602	1.08 (0.94-1.24)	0.264	0.94 (0.81-1.10)	0.444
Religion								
Catholic	1.00		1.00		1.00			
Other Christians	1.18 (1.04-1.34)	0.008	1.08 (0.95-1.22)	0.234	1.09 (0.97-1.23)	0.164		
Islam	0.73 (0.64-0.84)	<0.001	1.02 (0.86-1.21)	0.850	0.50 (0.43-0.58)	<0.001		
Traditionalist	1.10 (0.84-1.47)	0.49	1.65 (1.21-2.26)	0.002	0.77 (0.56-1.06)	0.117		
Others	1.03 (0.51-2.10)	0.92	0.90 (0.46-1.74)	0.747	0.72 (0.41-1.28)	0.261		
Household-level economical factors								
Wealth index								
Poorest	1.00		1.00		1.00		1.00	
Poorer	1.24 (1.11-1.39)	<0.001	1.14 (1.02-1.27)	0.021	1.22 (1.05-1.40)	0.007	1.11 (0.97-1.27)	0.116
Middle	1.57 (1.40-1.78)	<0.001	1.25 (1.10-1.43)	0.001	1.76 (1.51-2.06)	<0.001	1.24 (1.07-1.45)	0.004
Richer	2.07 (1.83-2.33)	<0.001	1.43 (1.24-1.65)	<0.001	1.99 (1.71-2.32)	0.001	1.13 (0.96-1.33)	0.135
Richest	2.69 (2.37-3.06)	<0.001	1.55 (1.31-1.84)	<0.001	2.47 (2.12-2.88)	<0.001	1.10 (0.93-1.30)	0.285
Frequency of reading newspapers or magazines								
Almost every day	1.00		1.00		1.00		1.00	
Not at all/less than once a week/at least once a week	2.29 (2.09-2.51)	<0.001	1.36 (1.25-1.49)	<0.001	1.63 (1.50-1.78)	<0.001	1.26 (1.16-1.37)	<0.001
Frequency of listening to radio								
Almost every day	1.00		1.00		1.00		1.00	
Not at all/less than once a week/at least once a week	0.61 (0.58-0.66)	<0.001	0.89 (0.83-0.96)	0.001	0.80 (0.74-0.86)	<0.001	0.90 (0.84-0.98)	0.009
Frequency of watching television								
Almost every day	1.00				1.00		1.00	
Not at all/less than once a week/at least once a week	0.52 (0.48-0.56)	<0.001			0.60 (0.54-0.65)	<0.001	0.91 (0.84-0.99)	0.046
Literacy								
Read whole sentences	1.00		1.00		1.00		1.00	
Can't read part/whole sentences	0.45 (0.42-0.49)	<0.001	0.79 (0.68-0.92)	0.002	0.51 (0.47-0.56)	<0.001	0.84 (0.73-0.97)	0.016
Community-level factors								
Place of residence								
Urban	1.00		1.00		1.00			
Rural	0.61 (0.55-0.68)	<0.001	0.88 (0.78-0.99)	0.031	0.74 (0.66-0.84)	<0.001		
Geographical region								
North-Central	1.00		1.00		1.00		1.00	
North-East	0.54 (0.45-0.64)	0.00	0.80 (0.66-0.97)	0.021	1.73 (1.41-2.12)	<0.001	2.27 (1.82-2.84)	<0.001
North-West	0.41 (0.51-0.73)	0.00	0.61 (0.50-0.75)	<0.001	0.68 (0.54-0.86)	0.001	1.16 (0.86-1.56)	0.331
South-East	0.57 (0.47-0.68)	0.00	0.53 (0.42-0.67)	<0.001	2.44 (2.01-2.96)	<0.001	2.04 (1.57-2.63)	<0.001
South-South	0.81 (0.68-0.96)	0.013	0.60 (0.50-0.72)	<0.001	2.32 (1.95-2.77)	<0.001	1.49 (1.21-1.83)	<0.001
South-West	0.95 (0.81-1.12)	0.563	0.66 (0.53-0.83)	<0.001	2.95 (2.49-3.50)	<0.001	3.14 (2.55-3.87)	<0.001
Ethnicity								
Ekoi	1.00		1.00		1.00		1.00	
Fulani	0.17 (0.13-0.23)	<0.001	0.21 (0.15-0.29)	<0.001	0.23 (0.12-0.30)	<0.001	0.30 (0.22-0.40)	<0.001
Hausa	0.22 (0.17-0.28)	<0.001	0.25 (0.19-0.35)	<0.001	0.14 (0.11-0.17)	<0.001	0.22 (0.16-0.29)	<0.001
Ibibio	0.42 (0.30-0.59)	<0.001	0.32 (0.23-0.45)	<0.001	0.40 (0.28-0.56)	<0.001	0.35 (0.24-0.50)	<0.001
Igala	0.71 (0.49-1.03)	0.072	0.41 (0.28-0.61)	<0.001	0.11 (0.06-0.17)	<0.001	0.14 (0.08-0.23)	<0.001
Igbo	0.34 (0.26-0.44)	<0.001	0.28 (0.21-0.37)	<0.001	0.53 (0.44-0.65)	<0.001	0.37 (0.29-0.47)	<0.001
Ijaw/Izon	0.31 (0.29-0.40)	<0.001	0.25 (0.18-0.37)	<0.001	0.62 (0.48-0.79)	<0.001	0.61 (0.48-0.79)	<0.001
Kanuri/Beriberi	0.29 (0.22-0.40)	<0.001	0.34 (0.24-0.46)	<0.001	0.30 (0.24-0.38)	<0.001	0.31 (0.23-0.41)	<0.001
Tiv	0.39 (0.29-0.52)	<0.001	0.29 (0.21-0.41)	<0.001	0.23 (0.18-0.29)	<0.001	0.39 (0.29-0.52)	<0.001
Yoruba	0.54 (0.42-0.70)	<0.001	0.34 (0.24-0.47)	<0.001	0.53 (0.44-0.64)	<0.001	0.26 (0.20-0.34)	<0.001
Others	0.44 (0.35-0.57)	<0.001	0.33 (0.26-0.43)	<0.001	0.41 (0.34-0.49)	<0.001	0.42 (0.34-0.52)	<0.001

AOR=Adjusted odds ratio; CI=Confidence interval; OR=Odds ratio; p=Level of significance

The study also revealed that majority of the respondents aged 15-49 years hinted they had heard of TB, and more than half of them had knowledge of the cure of TB. Less than half of the respondents had knowledge of TB being an airborne disease and that it could be transmitted from person to person during coughing or sneezing. These findings are broadly consistent with studies conducted in the Philippines, Ethiopia, China, and Viet Nam (13,17,23,24), which indicated that a large proportion of the population had heard of TB and also believed that TB is transmissible and curable. In another study on sandstone quarry workers in the desert parts of Rajasthan, other authors reported that a high percentage of the workers were aware that TB is a communicable disease (23).

Furthermore, our study indicated that male respondents were more aware of TB than their female counterparts. Also, compared to females, more males believed that TB could be transmitted from person to person through air during coughing or sneezing and that the disease is curable. These findings are broadly consistent with a study conducted by other authors (22) who stated that more females lacked knowledge about TB symptoms than males but females were more likely than males to seek healthcare relating to TB. Additionally, our findings are consistent with studies conducted in Sudan (25), India (21), and Ethiopia (22), which reported that the level of awareness among males about TB was significantly more than among females. The findings may also reflect differences in gender education in Nigeria where men are more likely to be educated than women (26).

The prevalence of each of the five outcome variables of interest (Table 3) was generally lower among non-literate respondents and higher among respondents with secondary or higher levels of education. Employed respondents and those who had access to the electronic media also showed a higher prevalence of each of the five outcome variables of interest. Our findings are consistent with studies by Okuonghae *et al.*, Mweemba *et al.*, and Mushtaq *et al.* (7,27,28), which concluded that high rates of illiteracy, low educational status, and unemployment status were common among TB patients. Also, a study conducted in Nigeria (7) observed that the lack of any formal education and non-access to the electronic media (TV and radio) were among the strongest predictors of high rates of TB cases in Benin-City in Nigeria. This finding also agrees with the results from two separate studies in Pakistan and Iraq (28,29), which indicated that the media play a vital role in patients’ knowledge about TB and, thus, highlight the need for TB health education programmes among non-literate households. The study in India (21) found only the radio, among the other media sources, to be associated with the correct knowledge of TB transmission.

This study showed a low prevalence of each of the five outcome variables of interest (Table 3) for rural and poor households. These findings are consistent with national household surveys conducted in the Philippines, Pakistan, and India (17,21,30), which indicated that higher knowledge about TB was observed among urban dwellers. This finding may reflect the fact that respondents who lived in rural areas and came from low socioeconomic backgrounds were more likely to feel embarrassed and stigmatized for having TB (28).

Results of our study showed that younger and unmarried respondents were less likely to report they had ever heard of TB and to report TB is spread through air during coughing or sneezing. A study in the Philippines (23) reported similar results. In Nigeria, as in most African countries, men and women eventually marry and, so, there is a strong link between age and marital status (31).

The prevalence of awareness about TB and the belief that TB is curable were found to be relatively lower among respondents from the North-Central geopolitical zone of Nigeria. Formal education might have played an important role in this finding as respondents from this zone had relatively lower levels of formal education compared to those from the other geopolitical zones of Nigeria (18). However, further research is needed to compare the prevalence of TB in each geopolitical region.

This study revealed that respondents who belonged to the Catholic faith reported a higher prevalence of each of the five outcome variables of interest (Table 3) compared to those from other religious denominations. These results may reflect the role of Roman Catholic Missionaries in building churches and hospitals to reduce the burden of the disease in Nigeria. For example, the Catholic church is one of the primary healthcare providers in Nigeria as they manage 31 TB screening and treatment centres across 16 states of the country (32).

A multiple binary logistic regression analysis method was used for showing that females, younger and never-married respondents were significantly associated with having ever heard of TB, reporting that TB is spread through air during coughing or sneezing and the belief that TB is curable. Respondents who watched television at least once a week or who did not watch television at all, poorest households as well as unemployed and uneducated respondents were also significantly associated with the outcome variables mentioned. Currently-married male respondents from rural areas, who were uneducated and unemployed and who watched television at least once a week or did not watch television at all, were associated with the report that they would keep a family member's TB secret and that TB is spread by sharing utensils. These results support similar findings that poverty, unemployment, no formal education, age, and gender were significantly associated with the knowledge of and attitude towards TB (11,23,27,29).

### Strengths and limitations of the study

One limitation of this study is that it only reported a ‘snapshot’ of the frequency of people's knowledge of and attitude towards TB since it had a cross-sectional design. Another limitation is that the study did not examine the relationship between HIV/AIDS and TB. Despite these limitations, the findings from this study would contribute to our understanding of the factors associated with the knowledge of and attitude towards TB, which may improve the quality and quantity of information dissemination about TB in Nigeria. Access to a large national survey, appropriate sampling method, and appropriate adjustment for sampling design, including sampling weight and a high response rate are important strengths of the survey.

### Conclusions

Our findings indicate that majority of the households had basic knowledge about TB but females, uneducated respondents, residents of poor households, and respondents who did not belong to the Catholic faith reported that they ever heard of TB, believed that TB could be cured, would want a family member's TB to be kept secret, and that TB is spread through coughing or sneezing and sharing utensils. The present study also showed that the literate people had a much better knowledge of and attitude towards TB than their non-literate counterparts. However, understanding of the factors associated with the knowledge of and attitude towards TB is considered the first stage of promoting healthcare-seeking behaviours, which may reduce the incidence of TB and will assist in the achievement of Millennium Development Goal 6. Finally, there is need for community mobilization and public education on TB in order to reduce the burden of TB in Nigeria.

## ACKNOWLEDGEMENTS

This analysis is a part of the first author's special project for a Master of Public Health degree with the Centre for Clinical Epidemiology and Biostatistics, University of Newcastle, NSW, Australia.
